# Characteristics of Design and Analysis of Ophthalmic Randomized Controlled Trials: A Review of Ophthalmic Papers 2020–2021

**DOI:** 10.1016/j.xops.2022.100266

**Published:** 2022-12-31

**Authors:** Ruiqi Dong, Gui-shuang Ying

**Affiliations:** 1 Department of Chemical and Biomolecular Engineering, University of Pennsylvania, Philadelphia, Pennsylvania; 2 Center for Preventive Ophthalmology and Biostatistics, Department of Ophthalmology, Perelman School of Medicine, University of Pennsylvania, Philadelphia, Pennsylvania

**Keywords:** Correlated Eye Data, Ophthalmic Clinical Trials, Statistical Analysis, Trial Design, GEE, generalized estimating equations, RCT, randomized clinical trial

## Abstract

**Objective:**

To evaluate the recent practice of design and statistical analysis of ophthalmic randomized clinical trials (RCTs).

**Design:**

Review of 96 ophthalmic RCTs.

**Methods:**

Two authors (R.D., G.S.Y.) reviewed primary result papers published from January 2020 through December 2021 in *Ophthalmology*, *JAMA Ophthalmology*, *American Journal of Ophthalmology*, and *British Journal of Ophthalmology*. Data were extracted and analyzed for the characteristics of design (1-eye design, 2-eye design, paired-eye design, and subject design), sample size and power, and statistical analysis for intereye correlation adjustment, missing data, and correction for multiplicity.

**Main Outcome Measures:**

Characteristics of trial design and statistical analysis.

**Results:**

Among 96 RCTs, 50 (52%) used 1-eye design, 21 (22%) 2-eye design, 10 (10%) paired-eye design, and 15 (16%) subject design. In 31 trials of 2-eye design or paired-eye design, 18 (58%) trials had suboptimal analysis of data from both eyes by analyzing data from 1 eye (n = 10), taking the average of 2 eyes (n = 2), analyzing 2 eyes separately (n = 1), ignoring intereye correlation (n = 3), or not specifying how 2-eye data were analyzed (n = 2), and 13 trials (42%) properly adjusted the intereye correlation by using the mixed-effects model (n = 6), paired *t* test (n = 5), generalized estimating equations (n = 1), or marginal Cox regression model (n = 1). Among 96 trials, 75 (78%) provided both sample size and statistical power estimation, and 16 (17%) trials described statistical test for sample size or power estimation. Missing data in primary outcome occurred in 86 (90%) trials with a median missing data rate of 8%, 32 (37%) trials applied statistical methods for missing data, including last value carried forward (n = 10), multiple imputation (n = 14), or other approaches (n = 8). Among 25 trials with > 2 arms, 16 (64%) corrected for multiplicity using the Bonferroni procedure (n = 8), Hochberg procedure (n = 2), Gatekeeping procedure (n = 2), or hierarchical procedure (n = 4). Among 16 trials with multiple primary outcomes, 4 (25%) corrected for multiplicity by the Bonferroni procedure.

**Conclusions:**

There are opportunities for improvement in the design and statistical analyses of ophthalmic trials, particularly in the aspects of adjustment for intereye correlation, missing data, and multiplicity. Continuing education in ophthalmology and vision research community may improve the quality of ophthalmic trials.

**Financial Disclosure(s):**

Proprietary or commercial disclosure may be found after the references.

Randomized clinical trials (RCTs) are the gold standard for evaluating the efficacy and safety of new treatments for eye diseases. Unlike clinical trials for diseases in other organs, ophthalmic trials may include treatment and follow-up of 1 or both eyes of a participant,^1^ and the treatment can be delivered ocularly or systemically; thus, both eyes of a participant can be in the same treatment group or in different treatment groups, complicating the trial design and statistical analysis. Therefore, an appropriate design of ophthalmic trials should consider whether 1 eye or both eyes of a participant are enrolled into the trial, and whether both eyes are assigned to the same treatment or different treatment. These considerations of enrollment of 1 or both eyes and their treatment assignment all impact the sample size/power estimation and statistical analysis of trial data because outcome measures from both eyes of a participant are usually positively correlated. The proper sample size/power estimation and statistical analyses of ophthalmic trials should adjust for the intereye correlation whenever ocular measures from both eyes of a participant are taken and included into statistical analyses.

Lee et al^2^ classified the study design of ophthalmic trials into the following 4 types: 1-eye design, 2-eye design, paired-eye design, and subject design. One-eye design includes only allocation of treatment and outcome assessment for 1 eye from each participant within the trial. On the contrary, 2-eye design includes allocations of treatment and outcome assessments for both eyes of each participant within the trial. Two-eye design is further classified as same-group design, different-group design, and mixed design. In the same-group design, both eyes of a participant are assigned to the same treatment group (e.g., systemic treatment or local ocular treatment). In the different-group design, 2 eyes of a participant are assigned to different local treatment groups. The paired-eye design is a special case of the different-group design, in which only 2 treatments are evaluated through within-participant comparison, and all participants are followed for outcomes in both eyes with 1 eye randomized to 1 treatment and the fellow eye randomized to another treatment. In mixed design, 2 eyes of a participant may be assigned to the same or different treatment. Subject design refers to trials with person-specific primary outcomes, such as quality of life or adverse effects of treatments.

Corresponding to the different ophthalmic trial designs, different sample size/power estimation and statistical analysis methods are necessary for the appropriate trial design and statistical analysis of trial data. For example, in the 2-eye design, depending on how 2 eyes of a participant are assigned to treatment groups, different statistical methods can be applied to adjust for the intereye correlation, and the impact of intereye correlation on the sample size/power estimation and statistical evaluation of treatment efficacy can also be different. Without adjusting for the intereye correlation, the precision of treatment efficacy can be overestimated if both eyes of a participant are in the same treatment group, whereas the precision of treatment efficacy can be underestimated if both eyes of a participant are in different treatment groups. Besides issues specific to ophthalmic trials, other issues common to any clinical trials, such as missing data and multiplicity of statistical comparisons, also need to be adequately addressed in ophthalmic clinical trials.^3^

To evaluate the practice pattern in the design and statistical analysis for ophthalmic trials, Lee et al^2^ reviewed 69 RCTs published in the 4 ophthalmology journals in 2009, including *Ophthalmology*, *JAMA Ophthalmology*, *American Journal of Ophthalmology*, and *British Journal of Ophthalmology*. They found substantial heterogeneity in the trial quality regarding proper trial design, sample size/power estimation, and statistical analysis.^2^

During the past decade, significant efforts have been made in the ophthalmology and vision research community to promote the best practice in trial design and statistical analysis, particularly on the consideration of 1 eye or both eyes for the trial, the adjustment of intereye correlation through review papers, tutorial papers,^1^^,^^2^^,^^4^, ^5^, ^6^, ^7^, ^8^, ^9^, ^10^, ^11^, ^12^, ^13^ and short courses at professional conferences (e.g., Association for Research in Vision and Ophthalmology). It is reasonable to assume that these activities may improve the trial design and statistical analysis of ophthalmic clinical trials. To evaluate the more recent practices of trial design and statistical analysis of ophthalmic trials, we reviewed primary results papers from ophthalmic RCTs published in 2020 and 2021 in 4 ophthalmology journals (*Ophthalmology*, *JAMA Ophthalmology*, *American Journal of Ophthalmology*, and *British Journal of Ophthalmology*). We report findings on the characteristics of design and statistical analysis of published ophthalmic trials and our evaluation of factors for their associations with practice patterns of trial design and statistical analysis. Through our review, we aimed to identify the areas of improvement for future ophthalmic trials.

## Methods

### Eligible RCTs

The first author (R.D.) manually screened for the primary result paper of RCTs published between January 1, 2020, and December 31, 2021, in 4 ophthalmology journals (*Ophthalmology*, *JAMA Ophthalmology*, *American Journal of Ophthalmology*, and *British Journal of Ophthalmology*). All eligible papers identified by the first author were confirmed by the senior author (G.S.Y.). Eligible papers were the first primary result paper (1 primary result paper per trial) reporting the main results for efficacy and safety of ≥ 1 interventions in a randomized controlled clinical trial. We excluded papers from nonrandomized studies, papers only reporting secondary outcomes of the ophthalmic trials, papers from the secondary analyses of data from the trials, and papers from the additional follow-up of trial participants beyond the primary end point. This study was conducted by reviewing published papers; it did not involve any human subjects. Thus, institutional review board approval and the Declaration of Helsinki do not apply.

### Data Extraction

The first author read the eligible papers and collected trial information about design characteristics and statistical analysis. All the relevant data were recorded in the Google form (Online Supplement, available at https://www.ophthalmologyscience.org). If the required information could not be found from the primary result paper, other resources were also searched, including supplemental documents for the primary result paper, the ClinicalTrials.Gov posting, the published trial protocol or design paper and other secondary papers from the same trial. The senior author confirmed all the data collected by the first author. The extracted data included the following:

1. Characteristics of the trial: type of intervention, type of eye disease, number of treatment arms, number of clinical centers, trial sponsor, country the trial was conducted in, nationality of the corresponding author, and length of participant follow-up for primary outcome assessment.
2. Characteristics of the trial design: type of trial design (1-eye design, 2-eye design, paired-eye design, and subject design). For 1-eye design, how the study eye was selected. For the 2-eye design, whether 2 eyes of a participant were assigned to the same or different treatment groups (e.g., same-group design, different-group design, mixture design).
3. Characteristics of outcome measures: data type (continuous, binary, survival, and count) and the number of primary and secondary outcomes. When the same measurement taken from different time points after randomization was indicated as different outcome measures in the primary outcome paper, we counted the measurement from different time points as multiple outcomes.
4. Sample size and statistical power: designed and actual sample size, planned statistical power, statistical method used for sample size, and power estimation.
5. Statistical analysis method for 2-eye data: for trials using 2-eye design, the information on statistical method used for analyzing the trial data, whether and how the intereye correlation was accounted for, and the statistical method for adjusting for the intereye correlation.
6. Missing data in primary outcome: The percentage with missing data in the primary outcome (calculated as the number of participants with missing data in the primary outcome divided by the total number of participants enrolled) and how the missing data were handled in the statistical analysis for the primary outcome.
7. Multiplicity of comparisons: correction for multiple comparisons because of multiple arms (> 2 arms) and multiple comparisons because of multiple primary outcomes (≥ 2) were assessed separately. Statistical methods for correcting these multiple comparisons were recorded.

### Statistical Analyses

Descriptive analyses were performed to summarize the characteristics of trial design and statistical analysis. Frequency and percentages were calculated for the categorical data. Mean (standard deviation), median (1st quartile and 3rd quartile), minimum, and maximum were calculated to summarize continuous data. We used the chi-square test to assess factors potentially associated with suboptimal trial design and statistical analysis, including adjustment for intereye correlation, missing data, and multiplicity. All statistical analyses were performed using the software Spyder (Python 3.8).

## Results

### Trial Characteristics

Eligible papers (n = 96) were the primary result paper reporting the RCTs published in 4 ophthalmology journals (*Ophthalmology*, *JAMA Ophthalmology*, *American Journal of Ophthalmology*, and *British Journal of Ophthalmology*) between January 1, 2020, and December 31, 2021 (Online Supplement, available at https://www.ophthalmologyscience.org). Among them, 25 (26%) papers were from *Ophthalmology*, 24 (25%) papers from *JAMA Ophthalmology*, 27 (28%) papers from the *American Journal of Ophthalmology*, and 20 (21%) papers from the *British Journal of Ophthalmology*. The characteristics of these 96 trials are summarized in Table 1. These 96 trials were designed for evaluating efficacy and safety of drug (54%), surgery procedure (22%), medical device (16%), education/training (4%), or ocular care model (4%) for a variety of eye diseases, including retina (25%), cornea (17%), cataract (15%), refractive error (15%), glaucoma (14%), and others (16%). Trials were funded by industry (31%), government (23%), university/hospital (13%), foundation (6%), > 1 sponsor (21%), or self-funding (6%). A majority (70%) of trials were 2 arms, 21% were 3 arms, and 9% were ≥ 4 arms. Most trials (83%) had 1 primary outcome, and the data type of primary outcome measure was continuous (68%), binary (29%), or time to event (3%). More than half (52%) were multicenter clinical trials. The trials were conducted internationally (18%), or in United States (29%), China (13%), India (7%), United Kingdom (5%), Japan (4%), Hong Kong (4%), Australia (3%), Austria (3%), and other countries (1%–2% each). Appropriately half (49%) of corresponding authors were from United States. The median (interquartile) length of follow-up for primary outcome assessment was 3 (1–12) months.

**Table 1:** Characteristics of the Ophthalmic Trials (N = 96)

Characteristics	Number	%
Journal of publication		
*Ophthalmology*	25	26%
*JAMA Ophthalmology*	24	25%
*American Journal of Ophthalmology*	27	28%
*British Journal of Ophthalmology*	20	21%
Type of intervention		
Drug	52	54%
Medical device	15	16%
Surgery	21	22%
Training/education	4	4%
Ocular care model	4	4%
Funding sponsor		
Government	22	23%
Industry	30	31%
Foundation	6	6%
University/hospital	12	13%
Multiple types	20	21%
No funding/self-funding	6	6%
Disease type		
Retina	24	25%
Cornea	16	17%
Cataract	14	15%
Refractive error	14	15%
Glaucoma	13	14%
Other	15	16%
Number of arms		
2	67	70%
3	20	21%
4	7	7%
5	2	2%
Number of primary outcome measures		
1	80	83%
2	11	11%
> 2	5	5%
Type of primary outcome measures		
Continuous	65	68%
Binary	28	29%
Time to event	3	3%
Number of clinical centers		
1	46	48%
> 1, ≤ 5	17	18%
> 5, ≤ 10	5	5%
> 10, ≤ 50	13	14%
> 50	15	16%
Country the trial was conducted in		
Multiple countries	17	18%
USA	28	29%
China	12	13%
India	7	7%
UK	5	5%
Japan	4	4%
Hong Kong	4	4%
Australia	3	3%
Austria	3	3%
Germany	2	2%
Korea	2	2%
Singapore	2	2%
France	1	1%
Thailand	1	1%
Netherland	1	1%
Italy	1	1%
Iran	1	1%
Denmark	1	1%
Brazil	1	1%
Nationality of the corresponding author		
USA	47	49%
China	11	11%
UK	6	6%
India	4	4%
Hong Kong	4	4%
Japan	3	3%
Australia	3	3%
Singapore	3	3%
Austria	2	2%
Germany	2	2%
Korea	2	2%
Italy	2	2%
France	1	1%
Thailand	1	1%
Netherland	1	1%
Iran	1	1%
Denmark	1	1%
Brazil	1	1%
Switzerland	1	1%
Length of follow-up for primary outcome (mos)		
≤ 1	28	29%
> 1, ≤ 6	30	31%
> 6, ≤ 12	26	27%
> 12	11	11%
Unknown	1	1%

UK = United Kingdom; USA = United States of America.

### Design Characteristics of Trials Involving 1 or Both Eyes of a Participant

A summary of the characteristics of trial design of these 96 trials is shown in Table 2. Fifty (52%) trials used 1-eye design. The study eye for the trial was the worse eye (11%), the better eye (2%), the first eye with the disease (7%), the randomly selected eye (5%), the right eye (1%), and the remaining 24 (25%) trials did not specify the selection of the study eye.

**Table 2:** Frequency Distribution of Type of Trial Design (N = 96)

Trial Design	Number	%
1-eye design	50	52
Worse eye	11	11
Better eye	2	2
First eye with disease	7	7
Randomly selected	5	5
Right eye	1	1
Not specified	24	25
		
2-eye design	21	22
Same-group design	17	18
Different-group design	2	2
Mixed design	2	2
Paired-eye design	10	10
Subject design	15	16

Overall, 21 trials (22%) used 2-eye design, including 17 trials with same-group design (e.g., both eyes in the same treatment group), 2 trials with different-group design (e.g., 2 eyes in different treatment groups), and 2 trials with mixed-group design (e.g., mixture of 2 eyes in the same or different treatment groups). Ten trials (10%) used paired-eye design to compare 2 treatments. The remaining 15 trials (16%) were subject design with participant-level primary outcomes, such as quality of life and nonocular test scores (academic test scores, King-Devik Test speed, etc.).

### Sample Size and Statistical Power of Clinical Trials

Among 96 eligible trials, 75 (78%) provided information on both sample size and statistical power estimation (Table 3), 6 (6%) trials only provided sample size estimation, whereas 4 (4%) trials only provided statistical power estimation, and 11 (12%) trials did not provide estimation of sample size nor statistical power. Among the 81 trials with sample size information, the median (1st quartile, 3rd quartile) of the designed sample size is 124 (50, 322), with range of 20 to 25 871 participants. Two trials^14^^,^^15^ with 20 participants used the efficient paired-eye design for ocular treatment of high myopia or glaucoma, whereas the trial^16^ with the largest sample size evaluated daily supplementation with vitamin D3 and marine ω-3 fatty acids for preventing the development or progression of age-related macular degeneration.

**Table 3:** Sample Size and Statistical Power of Trials (N = 96)

	Number	%
Providing both sample size and statistical power estimation		
No	21	23%
Only sample size	6	7%
Only statistical power	4	4%
Neither information	11	12%
Yes	75	78%
Designed sample size (Number of participants)		
Number of trials with sample size	81	
Median (1st quartile, 3rd quartile)	124 (50, 322)	
Min, max	20, 25 871	
Providing statistical power estimation		
No	17	18%
Yes	79	82%
80%	38	39%
> 80%, < 90%	10	10%
≥ 90%	31	32%
Statistical test for sample size estimation		
Not described	80	83%
Described	16	17%
Two-sample *t* test	7	7%
Paired *t* test	1	1%
Chi-square/Fisher exact test	3	3%
Analysis of variance	2	2%
Other	3	3%

Among all these 96 trials, 16 (17%) trials contained information on the statistical test that the estimation of sample size and statistical power was based on. Specifically, sample size/power estimation was based on *t* test (n = 7), paired *t* test (n = 1), chi-square test or Fisher exact test (n = 3), analysis of variance (n = 2), and other tests (n = 3). Among 79 trials with statistical power information, 38 (48%) trials were designed with 80% power, 10 (13%) trials with power > 80% and < 90%, and 31 (39%) trials with the power of ≥ 90%.

In our evaluation for potential factors associated with the provision of both sample size and statistical power estimation, we found that a lower percentage of trials providing both sample size and power was published in the *British Journal of Ophthalmology* (65%), in medical device trials (60%), with corresponding author in Asia (69%), government-funded trials (73%), and with continuous primary outcome (72%), yet none of these factors reached statistical significance (Table S4, available at https://www.ophthalmologyscience.org).

### Statistical Analysis for Trials Involving Both Eyes

Overall, 31 trials involved both eyes (2-eye design or paired-eye design). Their types of outcome measurements were continuous (n = 26, 84%), categorical (n = 3, 10%), or time to event (n = 2, 6%). Their statistical analysis methods for 2-eye data are summarized in Table 5. Among these 31 trials, 18 (58%) trials had suboptimal analysis of data from 2 eyes by only analyzing data from 1 eye (n = 10, 32%), taking the average of 2 eyes (n = 2, 6%), performing separate analysis for the left eye and right eye (n = 1), analyzing data from both eyes but ignoring intereye correlation (n = 3, 10%), or not specifying how 2-eye data were analyzed (n = 2, 6%). Only 13 (42%) trials analyzed data from both eyes and properly adjusted for intereye correlation by using the mixed-effects model (n = 6, 19%), paired *t* test (n = 5, 16%), generalized estimating equations (GEEs) (n = 1, 3%), or marginal Cox regression model (n = 1, 3%).

**Table 5:** Adjustment for Intereye Correlation in the Trials for Both Eyes of a Participant (N = 31)

	Number	%
Adjustment for intereye correlation in statistical analysis		
No	18	58%
Analyzed 1 eye data	10	32%
Analyzed the average of 2 eyes	2	6%
Separate analysis for left eye and right eye	1	3%
No adjustment	3	10%
Not specified	2	6%
Yes	13	42%
Mixed-effects model	6	19%
Paired *t* test	5	16%
Generalized estimating equations	1	3%
Marginal Cox regression model	1	3%

Potential factors associated with the proper statistical analysis of 2-eye data were evaluated (Table S6, available at https://www.ophthalmologyscience.org). Although a higher percentage of trials with proper statistical analysis was published in *Ophthalmology* (67%), in surgical trials (80%), in Europe (60%), and for binary primary outcome measures (67%), none of these associations were statistically significant.

### Missing Data in Primary Outcome

Among all 96 trials, 86 (90%) trials had missing data in the primary outcome, ranging from 0.3% to 65%, with 13 (15%) trials that had > 20% missing data in primary outcome (Table 7). The percentage of missing data in primary outcome was not significantly correlated with length of follow-up for primary outcome (Spearman correlation coefficient = 0.09, *P* = 0.38). Among these 86 trials with missing data, 32 (37%) trials applied statistical methods, including last value carried forward (n = 10, 12%), multiple imputation (n = 14, 16%), and other approaches (n = 8, 9%).

**Table 7:** Missing Data and Statistical Analysis Method for Missing Data

	Number		%
Missing data in primary outcome			
Absent	10		10%
Present	86		90%
% of missing data in primary outcome among trials with missing data			
> 0%, ≤ 5%	37		43%
> 5%, ≤ 10%	12		14%
> 10%, ≤ 20%	24		28%
≥ 20%	13		15%
Mean (SD)	12% (13%)		
Median (1st quartile, 3rd quartile)	8% (3%, 15%)		
Min, Max	0.3%, 65%		
Statistical method applied for missing data among trials with missing data			
Not applied	54		63%
Applied	32		37%
Last value carried forward	10		12%
Multiple imputation	14		16%
Other	8		9%

SD = standard deviation.

Among 86 trials with missing primary outcome data, we evaluated factors potentially associated with applying statistical methods for missing data (Table S8, available at https://www.ophthalmologyscience.org). The percentage of missing data in the primary outcome was not associated with the use of statistical methods for missing data (*P* = 0.92). In 37 trials with < 5% missing data, 14 (38%) applied statistical methods for missing data, whereas in 13 trials with > 20% missing data, only 3 (23%) trials applied statistical methods for missing data (*P* = 0.70). The trials published in *Ophthalmology* had the highest percentage (56%) of applying statistical methods for missing data, whereas trials published in the *American Journal of Ophthalmology* had the lowest percentage (9%, *P* = 0.006). Trials sponsored by the government (59%) had a higher percentage of applying statistical method for missing data than trials sponsored by industry (45%) and others (23%) (*P* = 0.02).

### Correction for Multiple Comparisons

Corrections of *P* values for multiple comparisons from multiple arms or multiple primary outcomes were evaluated (Table 9). Among 25 trials with > 2 arms, 16 (64%) trials controlled for multiplicity from multiple arms, including 8 trials using the Bonferroni correction, 2 trials using Hochberg procedure, 2 trials using Gatekeeping procedure, and 4 trials using hierarchical procedure. Among 16 trials having > 1 primary outcome, 4 (25%) trials corrected for multiplicity using the Bonferroni procedure, and the remaining 12 (75%) trials did not correct for multiple comparisons.

**Table 9:** Correction for Multiple Comparisons from Multiple Arms or Multiple Primary Outcomes

	Number	%
Number of trials with multiple arm trials	25	
Not corrected	9	36%
Corrected	16	64%
Statistical method for correcting multiple comparisons		
Bonferroni	8	32%
Hochberg	2	8%
Gatekeeping procedure	2	8%
Hierarchical procedure	4	16%
Number of trials with multiple primary outcomes	16	
Not corrected	12	75%
Corrected	4	25%
Statistical method for correcting multiple comparisons		
Bonferroni	4	25%

## Discussion

In this study, we reviewed a total of 96 primary result papers from ophthalmic RCTs for the trial design and statistical analysis. We found some trials are suboptimal in trial design and statistical analysis, particularly for the trials involving both eyes of a participant. The areas for improvement included the consideration for intereye correlation in sample size/power estimation and statistical analysis, handling of missing data in primary outcome, and correction for multiple comparisons.

Most eye diseases affect 2 eyes of a participant, so a good trial design should consider how to optimally use 2 eyes of a participant if both eyes are eligible for the trial. In our review, we found that about half (52%) of the trials used 1-eye design, 22% used 2-eye design, 10% used paired-eye design, and 16% used subject design. Similar to our finding, Lee et al^2^ reported 1-eye design in 48% trials, 2-eye design in 19% trials, paired-eye design in 13% trials, and subject design in 19% trials based on their review of 69 papers published in 2009 in the same 4 ophthalmology journals as our review.^2^ These results suggest that there was no substantial change in the design pattern of ophthalmic trials in the past decade.

The most common trial design is 1-eye design, in which only 1 eligible eye per participant was included into the trial; thus, intereye correlation is not required to be dealt with in the sample size estimation and statistical analysis. Among 50 trials using 1-eye design, a quarter of these trials chose the most severely affected eye as the study eye. The possible reasons for using the worse eye include the higher chance of achieving an improvement in outcome after the treatment, and the intention to reserve the better eye in case of failure of the treatment in the worse eye. It is concerning that there was no specification on how the study eye was selected in about half of 1-eye designed trials (24 of 50 1-eye trials). Although 1-eye design is appropriate in some ophthalmic trials for ocular diseases that are present in only 1 eye (e.g., ocular melanoma, amblyopia) and has the advantage in its simplicity by avoiding the need for the adjustment of intereye correlation, it is advantageous from a statistical perspective to use 2-eye design if both eyes of a participant are eligible. However, many other aspects of trial design must be considered in deciding 1-eye design versus 2-eye design and randomizing 2 eyes of a participant in the same treatment group versus different treatment group. Enrolling both eyes into the trial can be logistically complicated, particularly when 2 eyes are not enrolled or treated at the same time. The timing and frequency of treatments and follow-up may not be conducive to enrolling both eyes into the trial. Depending on the type of treatment, it could also be more difficult to mask the treatment groups in 2-eye design, potentially introducing bias in outcome assessment. Ethically, it may be more appropriate to treat each eye with a different treatment because if 1 intervention is better (or harmful), the participant will have received the better (or harmful) treatment in only 1 eye.^1^

In trials with 2-eye design, both eyes may receive the same or different treatments. Because ocular measures from 2 eyes of a participant are positively correlated, the nonindependence in the outcome measures from 2 eyes should be appropriately adjusted in the trial design and statistical analysis. Among 31 trials with 2-eye design or paired-eye design, only 42% trials made adjustment for intereye correlation. More than half of the trials (58%) either just analyzed data from 1 eye only (32%), or took the average of 2 eyes (6%), or performed 2 separate analyses of 2 eyes (3%), or analyzed data from both eyes but without adjustment of the intereye correlation (10%). When ocular measures from both eyes of a participant are highly correlated, analyzing data from 1 eye only or using the average of both eyes of a participant for analysis does not lead to loss of much statistical information and thus is appropriate, though less than ideal. However, analyzing data from both eyes without any adjustment of intereye correlation is incorrect. There is substantial improvement in the statistical analysis for trials with 2-eye design when compared with 62% found in Lee et al’s^2^ review of 13 trials with 2-eye design that did not adjust for the intereye correlation, 85% in Zhang and Ying’s^9^ review of 39 clinical studies, and 70% in Murdoch et al’s^4^ review of 23 clinical studies that did not adjust for intereye correlation when data from both eyes were analyzed. We believe that there is still room for improvement in the statistical analysis of 2-eye data. Collaborative efforts can be made in the ophthalmology and vision research community to improve the practice of statistical analysis for ocular data.

In 13 trials that appropriately adjusted for the intereye correlation, the primary statistical approach was mixed-effects model, initially developed for analyzing continuous data from longitudinal studies.^17^ Nevertheless, it can be applied for analyzing correlated continuous eye data.^5^^,^^7^ Other statistical methods for adjustment of intereye correlation were paired *t* test (19%) for paired-eye design, GEEs,^18^ and marginal Cox regression model.^19^ The mixed-effects model can be used for analyzing correlated continuous eye data.^5^^,^^7^ In contrast, the GEE can be used for analyzing correlated continuous eye data or binary eye data,^5^, ^6^, ^7^ whereas the marginal Cox regression model can be applied for analyzing the correlated time to event data. These model-based analysis approaches are very flexible in that they can be applied to the trial of the same-group design, different-group design, and mixed design, and to the trials with a mixture of unilateral or bilateral participants.^5^, ^6^, ^7^ By comparison, the simple paired *t* test can only be applied to analyze paired continuous data from the trial with 1 eye assigned to 1 treatment and the fellow eye in another treatment.

Power and sample size estimations are critical in the optimal design of trials. Our review found that a majority (78%) of trial primary result papers provided both sample size and power justification. However, only a small percentage of papers (17%) explicitly described the statistical test for sample size and power estimation, similar to the 18% reported in Lee et al’s^2^ study. Among 16 papers that described statistical methods for sample size estimation, *t* test and chi-square test were the most common approaches, which did not account for the intereye correlation. Proper sample size and power estimation for ophthalmic trials should deliberate whether both eyes of a participant are included in the trial, and whether both eyes are in the same treatment group or not, and properly account for the intereye correlation if ocular measures are taken and analyzed for both eyes of a trial participant. There have been several published papers that described various approaches of sample size and statistical power estimation for ophthalmic trials involving 1 or both eyes.^20^, ^21^, ^22^, ^23^ The impact of intereye correlation on sample size and power of trials with 2-eye design depends on whether 2 eyes of a participant are assigned to the same treatment group or not. If 2 eyes of a participant are in the same treatment group (e.g., systemic treatment), ignoring the intereye correlation will underestimate the sample size (or overestimate the power). If 2 eyes of a participant are in 2 different treatment groups, ignoring the intereye correlation will overestimate the sample size (or underestimate the power). For example, for designing a trial to detect difference in the ocular event rate of 20% in treated group and 30% control group with 80% statistical power at type 1 error rate of 0.05, if 2 eyes of a participant are in the same treatment group, the total sample size will be 294 participants when the intereye correlation is ignored (e.g., assuming independence between 2 eyes), but will increase to 442 participants if the moderate intereye correlation (0.50) is considered. If the paired design is used (i.e., 1 eye in the treated group and the fellow eye in the control group), the sample size will be 294 participants if intereye correlation is not considered; it will decrease to 142 participants if moderate intereye correlation (0.50) is considered.

Missing data are very common in clinical trials for a variety of reasons, such as the inability or unwillingness to complete follow-up visits for outcome evaluation.^24^ Our review found that 90% of trials had missing data in primary outcome measure, with 15% trials that had > 20% missing data in primary outcome. Missing data can not only reduce the statistical power but can also introduce bias in the estimation of the treatment effect if missing data are not at random. Therefore, prudent trial design and trial conduct to prevent missing data and the proper statistical handling of missing data are essential. Modern statistical analysis tools, such as maximum likelihood estimation, multiple imputation, Bayesian methods, and methods based on GEE, can reduce the potential bias arising from missing data.^24^ Even though many statistical analysis approaches for missing data are available, our review found that only 37% of trials applied missing data methods with multiple imputation most commonly used. In our evaluation for factors associated with applying statistical methods for missing data, we found that government-funded trials (*P* = 0.02) and trials published in *Ophthalmology* (*P* = 0.006) had a significantly higher percentage of applying statistical methods for missing data, which could be because of the strict scientific review process for the government-funded trials or stricter-peer review process for trials published in *Ophthalmology*.

It is well-known that multiple statistical comparisons in clinical trials can inflate type 1 error (e.g., incorrectly claiming a new treatment is efficacious when in truth it is not) for evaluating the efficacy of a new treatment; thus, corrections for multiplicity to maintain the desirable (5%) type 1 error rate are necessary. Multiple comparisons in clinical trials can arise from multiple treatment arms (> 2) or multiple outcomes (≥ 2). The guidelines for clinical trials recommend that multiplicity corrections should be performed to control the overall type 1 error in most circumstances, particularly for confirmatory phase III trials.^25^, ^26^, ^27^, ^28^ Lack of correction can lead to the overstatement of statistical significance of the treatment effect.^26^ Our review found that among 25 trials with > 2 treatment arms, multiplicity was corrected in 64% of them, and the most commonly used method was the Bonferroni correction. However, in 16 trials with multiple primary outcomes, only 4 trials were corrected for the multiple comparisons from multiple outcomes, all by the Bonferroni method. This review suggested that the practice of multiple comparisons needs improvement in ophthalmic trials.

In this study, we performed comprehensive review of the trial design and statistical analysis for 96 ophthalmic RCTs published between January 1, 2020, and December 31, 2021, in 4 ophthalmology journals (*Ophthalmology*, *JAMA Ophthalmology*, *American Journal of Ophthalmology*, and *British Journal of Ophthalmology*). Our selection of these journals is mainly to compare our findings with those from Lee et al’s^2^ review of 69 trials published in these 4 ophthalmology journals in 2009. Because these 4 journals are top ophthalmology journals, findings from this review may not be generalizable to the ophthalmic trials published in other ophthalmology journals. Similar review of trials published in other ophthalmology journals will be performed in the future.

In conclusion, our review of 96 primary result papers from randomized ophthalmic trials identified areas for improvement in the ophthalmic trial design and statistical analysis. For trials involving 2 eyes of a participant, adjustment for intereye correlation should be made in the sample size estimation and statistical analysis. The statistical tools (such as multiple imputation) for handling missing data in primary outcome measures can be more frequently applied in statistical analysis. Correction for multiple comparisons from multiple arms or multiple primary outcomes should be considered to control type 1 error rate. Education in the ophthalmology and vision research community through workshops, short course, and tutorial papers may help improve the quality of future ophthalmic trials.
