# Supplementary material for: Characteristics of Design and Analysis of Ophthalmic Randomized Controlled Trials: A Review of Ophthalmic Papers 2020–2021
Source: Ophthalmol Sci. 2022 Dec 31;3(2):100266. doi: 10.1016/j.xops.2022.100266 (PMC9926296; doi:10.1016/j.xops.2022.100266)
Supplement: Online Table S4 [file mmc1.pdf]

Online supplement Table S4: Factors Potentially Associated with Provision of both Sample Size and Statistical Power Estimation in Trials (N=96)

Factors	n	Yes (%)*	P-value
**Journal**			0.45
Ophthalmology	25	20 (80%)	
JAMA Ophthalmology	24	20 (83%)	
American Journal of Ophthalmology	27	22 (81%)	
British Journal of Ophthalmology	20	13 (65%)	
**Type of Intervention**			0.16
Drug	52	44 (85%)	
Medical Device	15	9 (60%)	
Surgery	21	15 (71%)	
Other	8	7 (88%)	
**Nationality of Corresponding Author**			0.34
Asia	29	20 (69%)	
Europe	16	14 (88%)	
North America	47	37 (79%)	
Other	4	4 (100%)	
**Funding Sponsor**			0.68
Government	22	16 (73%)	
Industry	30	23 (77%)	
Other	44	36 (82%)	
**Data Type**			0.12
Continuous	65	47 (72%)	
Binary	28	25 (89%)	
Time to Event	3	3 (100%)	

*"Yes" means both sample size and statistical power estimation was provided.
