# Supplementary material for: Characteristics of Design and Analysis of Ophthalmic Randomized Controlled Trials: A Review of Ophthalmic Papers 2020–2021
Source: Ophthalmol Sci. 2022 Dec 31;3(2):100266. doi: 10.1016/j.xops.2022.100266 (PMC9926296; doi:10.1016/j.xops.2022.100266)
Supplement: Online Table S6 [file mmc2.pdf]

Online supplement Table S6: Factors Potentially Associated with Appropriate Adjustment of Inter-eye Correlation for Two-eye Design or Paired-eye Design (N=31)

**Factors**		**n**	**Yes (%)***	**P-value**
**Journal**				0.55
	Ophthalmology	6	4 (67%)	
	JAMA Ophthalmology	4	1 (25%)	
	American Journal of Ophthalmology	13	5 (38%)	
	British Journal of Ophthalmology	8	3 (38%)	
**Type of Intervention**				0.11
	Drug	18	7 (39%)	
	Medical Device	4	2 (50%)	
	Surgery	5	4 (80%)	
	Other	4	0 (0%)	
**Nationality of Corresponding Author**				0.69
	Asia	13	5 (38%)	
	Europe	5	3 (60%)	
	North America	12	5 (42%)	
	Other	1	0 (0%)	
**Funding Sponsor**				0.46
	Government	6	2 (33%)	
	Industry	10	3 (30%)	
	Other	15	8 (53%)	
**Data Type**				0.63
	Continuous	26	10 (38%)	
	Binary	3	2 (67%)	
	Time to Event	2	1 (50%)	

*"Yes" means adjustment for inter-eye correlation was made.
