# Supplementary material for: Characteristics of Design and Analysis of Ophthalmic Randomized Controlled Trials: A Review of Ophthalmic Papers 2020–2021
Source: Ophthalmol Sci. 2022 Dec 31;3(2):100266. doi: 10.1016/j.xops.2022.100266 (PMC9926296; doi:10.1016/j.xops.2022.100266)
Supplement: Online Table S8 [file mmc3.pdf]

Online supplement Table S8: Factors Potentially Associated with Applying Statistical Method for Missing Data in Primary Outcome (N=86)

Factors		n	Yes (%)*	P-value
**% of missing data**				0.70
	≤ 5%	37	14 (38%)	
	>5%, ≤ 10%	12	5 (42%)	
	>10%, ≤20%	24	10 (42%)	
	≥20%	13	3 (23%)	
**Journal**				**0.006**
	Ophthalmology	25	14 (56%)	
	JAMA Ophthalmology	23	11 (48%)	
	American Journal of Ophthalmology	22	2 (9%)	
	British Journal of Ophthalmology	16	5 (31%)	
**Type of Intervention**				0.72
	Drug	46	18 (39%)	
	Medical Device	14	5 (36%)	
	Surgery	18	5 (28%)	
	Other	8	4 (50%)	
**Nationality of Corresponding Author**				0.61
	Asia	24	7 (29%)	
	Europe	16	5 (31%)	
	North America	43	19 (44%)	
	Other	3	1 (33%)	
**Funding Sponsor**				**0.02**
	Government	17	10 (59%)	
	Industry	29	13 (45%)	
	Other	40	9 (23%)	
**Data Type**				0.68
	Continuous	56	19 (34%)	
	Binary	28	12 (43%)	
	Time to Event	2	1 (50%)	

*”Yes” means statistical methods for dealing with missing primary outcome data were applied.
